# *Tibouchina granulosa* Leaves Present Anti-Inflammatory Effect

**DOI:** 10.3390/ph15121458

**Published:** 2022-11-24

**Authors:** Carolina Carvalho Guilhon, Alan Silva Minho, Marc Pouliot, Fabio Boylan, Patricia Dias Fernandes

**Affiliations:** 1 Laboratório de Farmacologia da Dor e da Inflamação, Programa de Pesquisa em Descoberta de Fármacos, Instituto de Ciências Biomédicas, Universidade Federal do Rio de Janeiro, Rio de Janeiro 21941-902, Brazil; 2 Centre de Recherche du CHU de Québec, Université Laval, Québec, QC G1V 4G2, Canada; 3 School of Pharmacy and Pharmaceutical Sciences and Trinity Biomedical Sciences Institute, Trinity College Dublin, 2 Dublin, Ireland

**Keywords:** *Tibouchina granulosa*, anti-inflammatory activity, hispiduloside, cytokines

## Abstract

The ethanol extract (EE) prepared from the leaves of *Tibouchina granulosa*, and its fraction in ethyl acetate (fEA) were evaluated concerning their capacity to reduce inflammation in different experimental models. fEA was also studied concerning its chemical constituents. EE and fEA were assayed for their anti-inflammatory potential, using formalin-induced licking behavior and carrageenan-induced inflammation into the subcutaneous air pouch (SAP) models. Reduction in polymorphonuclear cells (PMN) activation was performed in freshly isolated PMN. Chromatographic analysis of fEA was performed by HPLC-DAD. Hispiduloside was isolated as the main constituent in fEA, and its quantity was estimated to be 39.3% in fEA. EE (30 mg/kg) significantly reduced the second phase of formalin-induced licking. fEA demonstrated a reduction in leukocyte migration into the SAP. EE and fEA drastically reduced cytokines (TNF-α, IL-1β, and IFN-γ), nitric oxide (NO) production, in vitro PMN migration induced by C5a and IL-8, and TNF-α and IL-1β gene expression. Taken together, our data indicate that either ethanol extract or its fEA fraction from leaves of *T. granulosa* present an anti-inflammatory effect, contributing to the pharmacological and chemical knowledge of this species and confirming the rationale behind its traditional use.

## 1. Introduction

Natural products and their special metabolites have been the most successful source of new potential drugs since less than 10% of the world’s biodiversity has been evaluated for potential biological activity [1]. Historically, natural products have been used as medicinal therapy since ancient times for the treatment of many diseases and illnesses. Society searches in nature for aid for their basic needs, including medical treatment [2]. Due to their vast structure diversity, natural products derived from medicinal plants and/or microorganisms provide “privileged scaffolds” and have thus been significant contributors to drug development [3].

Inflammation is a beneficial host response to a foreign challenge or tissue injury that leads ultimately to the restoration of tissue structure and function. In other words, inflammation is an immunological defense mechanism elicited in response to mechanical injuries, such as burns, microbial infections, allergens, and other noxious stimuli [4]. This response requires innate immunity and, in some cases, an adaptive immune response, which are the two main integral components of the host’s defense system. Innate immunity not only acts as the first line of defense against noxious material, but after recognition of an appropriate stimulus, it provides the necessary signals to instruct the adaptive immune system to mount a response [5,6]. In turn, the adaptive response relies on the innate immune system to provide the necessary effectors, in the form of phagocytes and granulocytes, to deal with the initiating stimulus. However, prolonged inflammation can cease to be a beneficial event, and it contributes to the pathogenesis of many disease states [4]. It develops in the classical signs of redness, swelling, heat, and hyperalgesia. These symptoms result from the action of inflammatory agents, such as bradykinin, serotonin, histamine, prostaglandins, leukotrienes, and nitric oxide, which can be originated locally or from cells that infiltrate on the site of injury [7].

Non-steroidal anti-inflammatory drugs (NSAIDs) are among the most widely prescribed therapeutics, primarily for the treatment of pain and inflammation. However, the long-term clinical usage of NSAIDs is associated with significant side effects, such as gastrointestinal lesions, bleeding, and peptic ulcers [8]. As an alternative, plant-based medicines are increasingly receiving more attention in the therapeutics market share due to their mild action and fewer adverse effects profile [2].

*Tibouchina granulosa*, popularly known as ‘quaresmeira’, is a member of the Melastomataceae family and is an ornamental tree widely distributed in the Brazilian Atlantic Forest. Some studies have described anti-inflammatory, diuretic, depurative, and antioxidant effects for species belonging to this family, such as *Osbeckia parvifolia* Arn., *Tibouchina asperior* Cogn. and *Clidemia rubra* Mart [9,10,11]. There are descriptions of the traditional use of *Tibouchina* species as an antioxidant [12], anticancer [13], protection against microorganisms such as endodontic bacteria (*A. naeslundii, P. gengivalis, B. fragilis, P. nigrescens*) [14], fungi (i.e., *C. albicans, A. fumigatus,* and *F. solani*) [15,16], and leishmania [17,18], and antinociceptive [19]. Previous work from our group demonstrated that the aqueous extract of *T. granulosa* leaves presented a significant anti-inflammatory effect, decreasing cell migration and inflammatory mediators’ production [20]. However, it is still unknown whether extracts prepared from the leaves could present anti-inflammatory activity as well as inhibiting the migration of human isolated blood cells in vitro. These questions led us to question whether an ethanol extract and a fraction obtained from the leaves of *T. granulosa* could present those effects. In this regard, the present study aimed to evaluate the anti-inflammatory properties of the ethanol extract (EE) and fraction in ethyl acetate (fEA) obtained from the leaves of *T. granulosa* and tried to correlate the potential activity with the presence of hispiduloside in both extracts.

## 2. Results

### 2.1. Chemical Analysis

**Figure 1:**
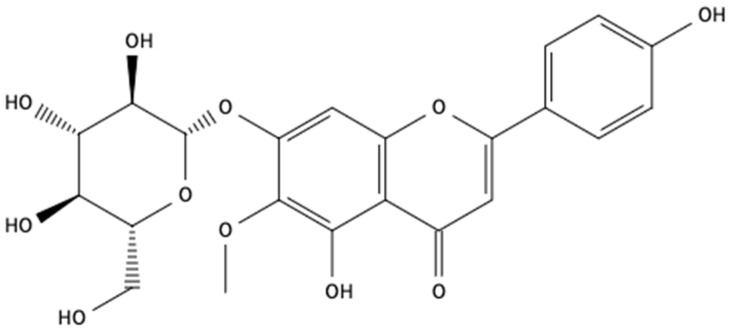
Chemical structure of hispiduloside (hispidulin 7-O-D-glucoside).

**Figure 2:**
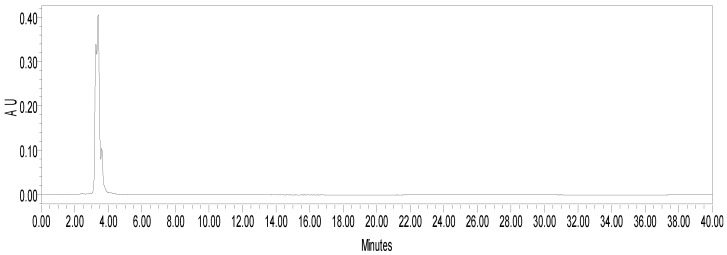
HPLC-DAD chromatogram at 355 nm, using a mobile phase gradient and a reversal phase C18 column, showing hispiduloside at retention time 3.897 min.

Column chromatography of fEA led to the isolation of hispiduloside (Figure 1) from fractions 114–128, eluted with ethyl acetate: methanol 20 and 30%. They were combined, further purified by preparative TLC, and the pure compound was isolated, and its structure was confirmed using ^1^H- and ^13^C-NMR followed by comparison with previously published data [21]. fEA was analyzed by HPLC (Figure 2), and hispiduloside (3.897 min) was further quantified in the plant at 0.02% (*w*/*w*). There was no hispiduloside left for the pharmacological assays.

### 2.2. T. granulosa EE and fEA Reduced Paw Licking Behavior Induced by Formalin Injection

**Figure 3:**
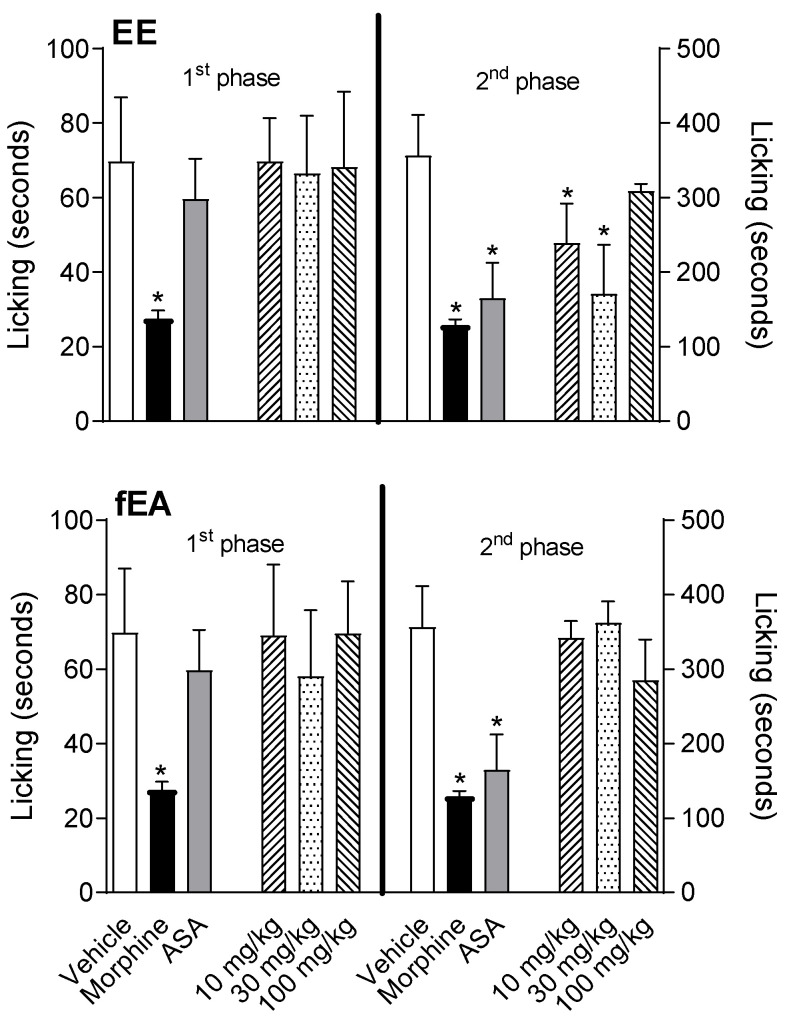
Effect of the ethanol extract (EE) or fraction in ethyl acetate (fEA) from leaves of *T. granulosa* in the formalin-induced licking behavior. The animals were pre-treated with vehicle, morphine (2.5 mg/kg), ASA (200 mg/kg), EE or fAE (10, 30 or 100 mg/kg), 60 min before the intra-plantar injection of formalin (2.5% *v*/*v*). The results are presented as mean ± S.D. of the time (seconds) that animals kept licking their formalin-injected paws (*n* = 6). The statistical significance (* *p* < 0.05) had been calculated comparing the groups pre-treated with EE, fEA, morphine, or ASA with the group pre-treated with vehicle, using ANOVA followed by post-test of Newman–Keuls.

The intraplantar injection of formalin (2.5% *v*/*v*) triggered a nociceptive response, divided into two different phases. The first one (neurogenic phase) is known to happen in the first 5 min after the formalin injection, and the second phase (inflammatory) happens between fifteen and thirty minutes after the injection. The results presented in Figure 3 show the effect of the EE and fEA (10, 30, and 100 mg/kg). In the first phase of the test, formalin induced a licking time of 69.8 ± 15.4 s (vehicle-treated group). The acetylsalicylic acid (ASA, 200 mg/kg)-treated group (positive control group) did not present a reduction in licking time (to 59.7 ± 9.7 s), while morphine significantly reduced the response (27.6 ± 4.9 s). Neither extract nor fraction had any effect in the first phase of the model. All groups presented similar results when comparing with the vehicle-treated group. When evaluating the second phase, it was observed that animals pre-treated with the vehicle developed a licking time response of 336.9 ± 47.9 s. ASA and morphine significantly reduced the response in 51.2% and 61.7% (164.5 ± 43.5 s and 129 ± 16.6 s), respectively. fEA did not show any effect in the second phase of this test. Surprisingly, EE and fEA had no effects at their higher doses (100 mg/kg).

### 2.3. T. granulosa fEA Reduced Leukocytes Migration into Subcutaneous Air Pouch (SAP)

**Figure 4:**
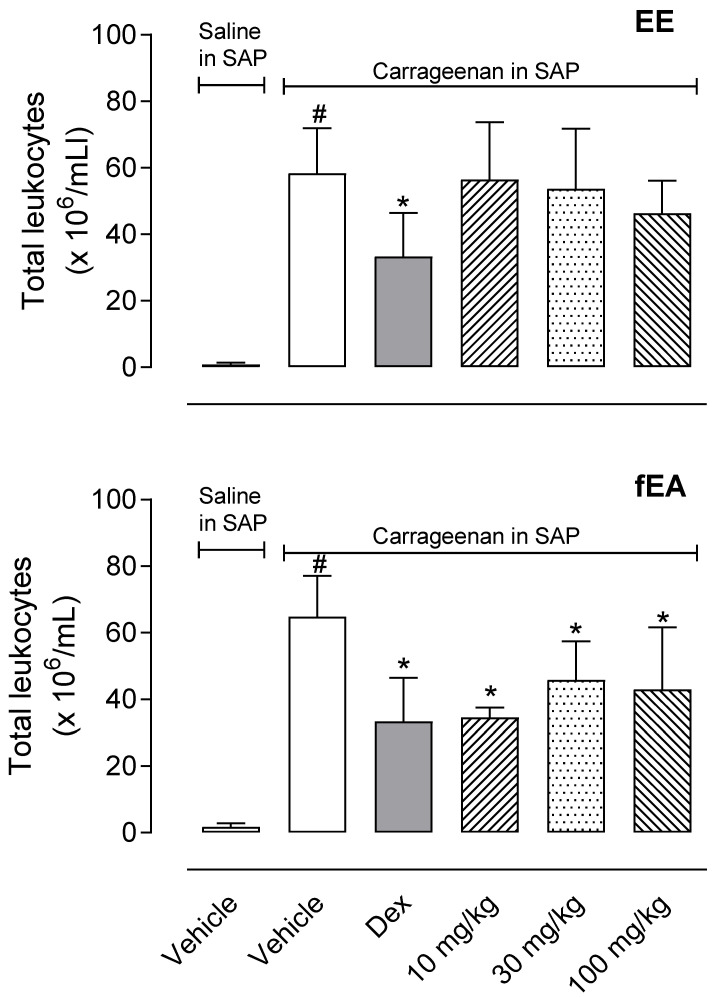
Effect of the ethanol extract (EE) and fraction in ethyl acetate (fEA) from leaves of *T. granulosa* in leukocyte migration into the subcutaneous air pouch. The animals were pre-treated with vehicle, dexamethasone (Dex, 0.5 mg/kg, i.p.), EE or fEA (10, 30, or 100 mg/kg), 60 min before the injection of carrageenan (1%) or saline in the SAP. The results are presented as mean ± S.D. (*n* = 7). The statistical significance was calculated comparing the groups pre-treated with EE, fEA, or dexamethasone with the group pre-treated with the vehicle, using ANOVA followed by the post-test of Newman–Keuls. # *p* < 0.05 when comparing the vehicle-treated group receiving carrageenan in SAP with the vehicle-treated group that received saline in SAP; * *p* < 0.05 when comparing dex-, EE-, or fEA-treated groups that received carrageenan in SAP with the vehicle-treated group receiving carrageenan in SAP.

Carrageenan induced a 58-fold increase in the number of cells that migrated to the SAP (0.9 ± 0.5 × 10^6^ cells/mL in saline-injected SAP versus 58.4 ± 13.5 × 10^6^ cells/mL in carrageenan injected SAP). When animals were pre-treated with dexamethasone, the leukocyte’s migration was decreased by 45%. Although EE did not affect cell migration, we could observe that fEA significantly reduced the number of leukocytes in the cavity (Figure 4).

### 2.4. T. granulosa EE and fEA Reduced Inflammatory Mediators’ Productions in SAP

**Figure 5:**
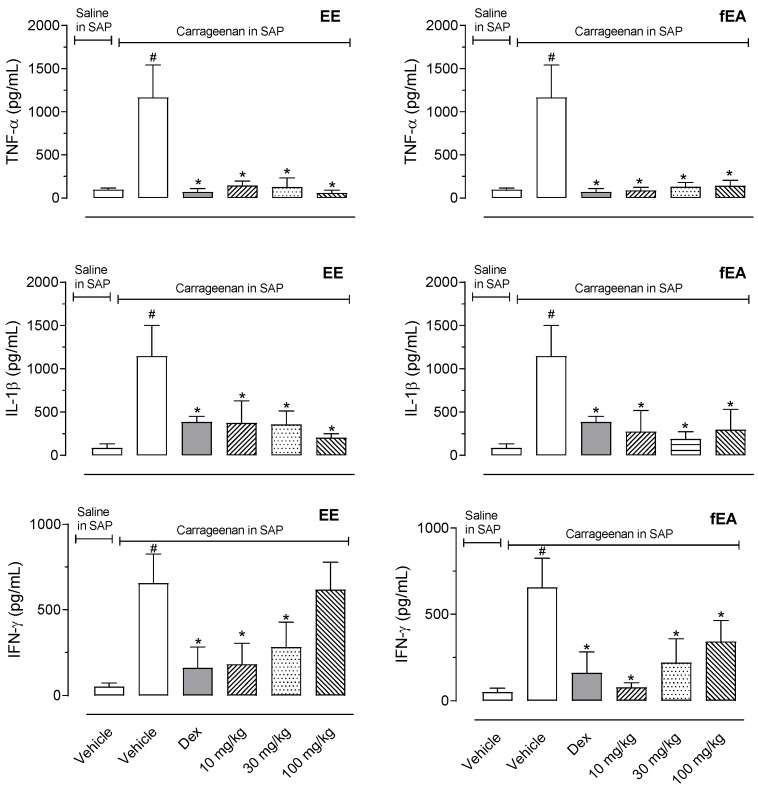
Effect of the ethanol extract (EE) and fraction in ethyl acetate (fEA) from leaves of *T. granulosa* in TNF-α, IL-1β, and IFN-γ production in the SAP. The animals were pre-treated with vehicle, dexamethasone (Dex, 0.5 mg/kg, i.p.), EE or fEA (10, 30 or 100 mg/kg), 60 min before the injection of carrageenan (1%) or saline into SAP. The results are presented as mean ± S.D. (*n* = 7). The statistical significance was calculated comparing the groups pre-treated with EE, fEA, or dexamethasone with the group pre-treated with the vehicle, using ANOVA followed by the post-test of Newman–Keuls. # *p* < 0.05 when comparing the vehicle-treated group receiving carrageenan in SAP with the vehicle-treated group that received saline in SAP; * *p* < 0.05 when comparing dex-, EE- or fEA-treated groups that received carrageenan in SAP with the vehicle-treated group receiving carrageenan in SAP.

Pre-treatment with EE or fEA provided an almost complete reduction in the cytokines measured in the exudate. EE and fEA presented an important inhibitory effect, completely abolishing TNF-α and IL-1β production (Figure 5).

### 2.5. T. granulosa EE and fEA Reduced the Production of Nitric Oxide in SAP

**Figure 6:**
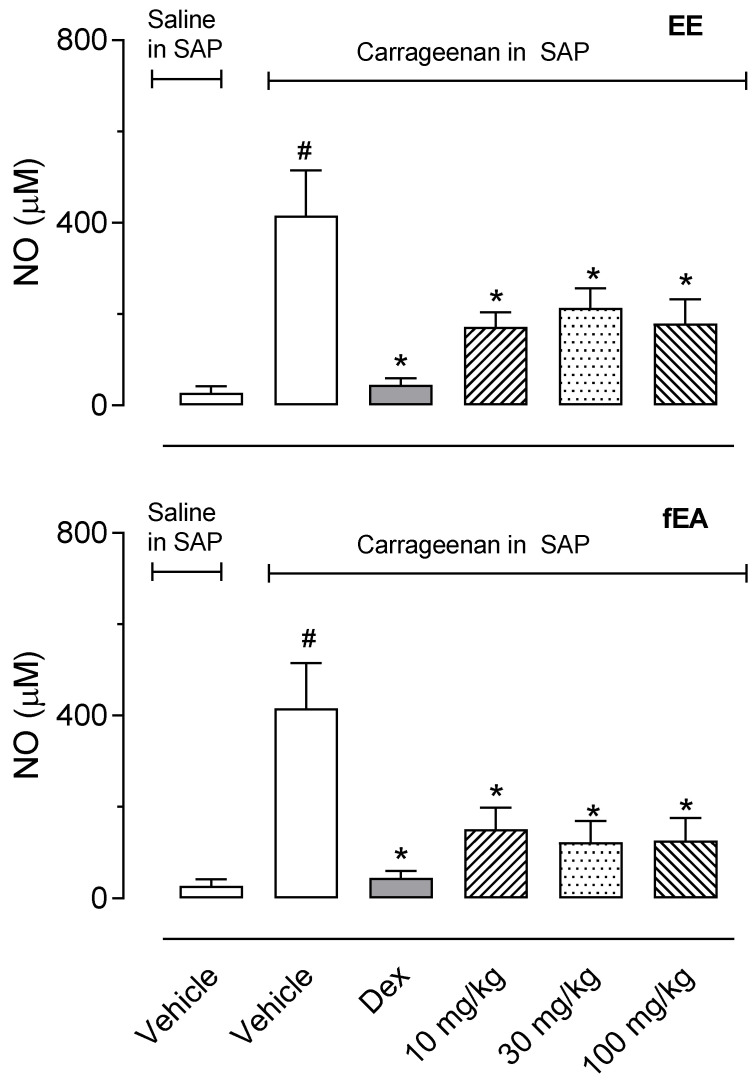
Effect of the ethanol extract (EE) and fraction in ethyl acetate (fEA) from leaves of *T. granulosa* in nitric oxide (NO) accumulated in the exudate of SAP. The animals were pre-treated with vehicle, dexamethasone (Dex, 0.5 mg/kg, i.p.), EE or fEA (10, 30 or 100 mg/kg), 60 min before the injection of carrageenan (1%) or saline into the SAP. The results are presented as mean ± S.D. (*n* = 7). The statistical significance was calculated comparing the groups pre-treated with EE, fEA or dexamethasone with the group pre-treated with the vehicle, using ANOVA followed by the post-test of Newman–Keuls. # *p* < 0.05 when comparing the vehicle-treated group receiving carrageenan in SAP with the vehicle-treated group that received saline in SAP; * *p* < 0.05 when comparing dex-, EE- or fEA-treated groups that received carrageenan in SAP with the vehicle-treated group receiving carrageenan in SAP.

To further evaluate the effect of *T. granulosa* in the inflammatory phenomena, we analyzed the effects of EE and its fraction against the production of nitric oxide (NO) by leukocytes infiltrated in the SAP. Data shown in Figure 6 demonstrate that EE and fEA significantly decreased NO production. Nevertheless, fEA decreased the NO production by 63.6, 70.5, and 69.6%, suggesting fEA to be the most potent in reducing NO production (Figure 6).

### 2.6. T. granulosa EE and fEA Decreased Polymorphonuclear Neutrophils (PMNs) Migration Induced by IL-8 and C5a

Given the results obtained in the SAP demonstrating that EE and fraction did significantly reduce cell migration, we decided to further evaluate if a similar effect could also be observed when using purified human polymorphonuclear cells (PMNs).

The human freshly isolated PMNs were stimulated with well-known chemotaxis factors, C5a and IL-8, and incubated with different concentrations of EE or fEA, the extract and fraction that presented the better results in inhibiting in vivo leukocyte migration.

**Figure 7:**
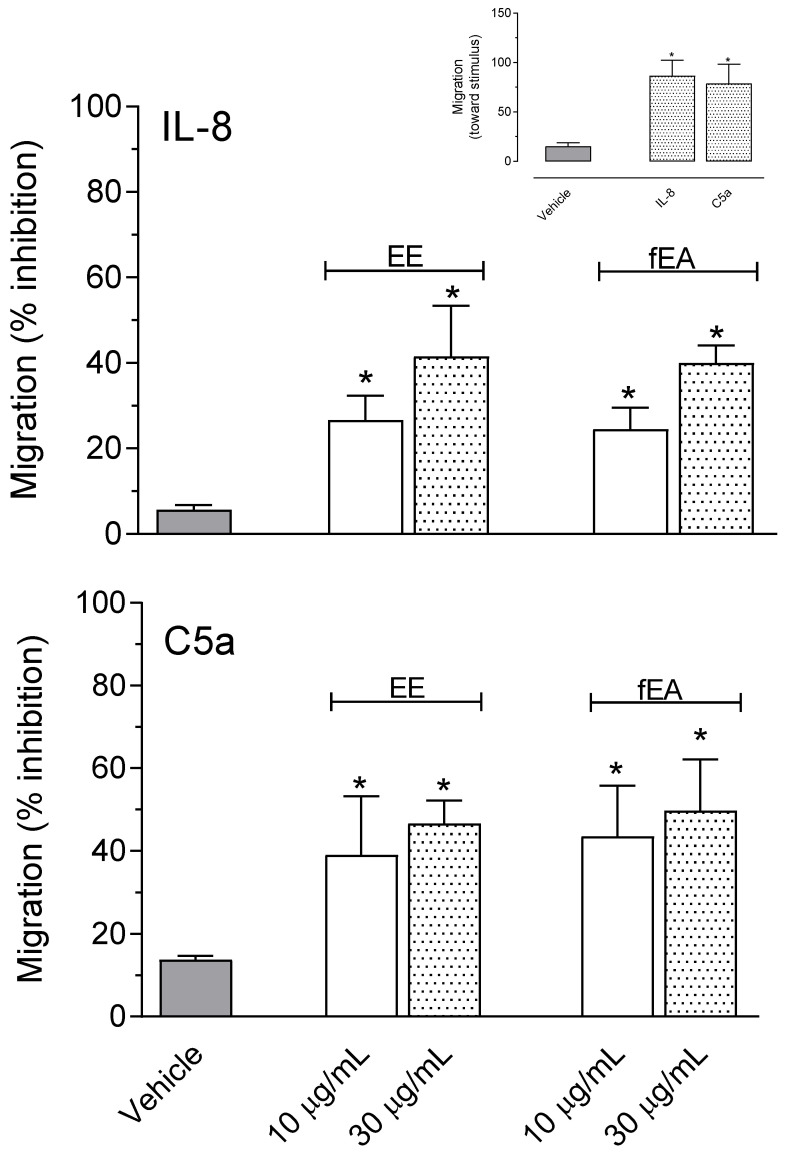
Effect of EE and fEA in reducing freshly isolated human polymorphonuclear cells (PMN) stimulated with IL-8 or C5a. PMNs were preincubated with EE or fEA (10 and 30 µg/mL) and after that activated by IL-8 (10^−7^ M) or C5a (10^−8^ M). The insert shows the percentage of PMN that migrated toward saline, IL-8 or C5a as stimuli. The statistical significance was calculated using analyses of variance ANOVA followed by the post-test of Newman–Keuls. * *p* < 0.05 when comparing the cells that received EE or fEA with the vehicle or when comparing the cells that received IL-8 or C5a as stimuli with group receiving saline as stimuli (insert).

As can be seen in Figure 7, both EE and fEA, at any of the tested concentrations (10 and 30 µg/mL), significantly reduced the migratory capacity of PMN stimulated by either of the two chemotactic stimuli, IL-8 or C5a. Both stimuli induced 86.4% and 78.8% PMN migration, respectively. Saline alone did induce only 15.3% of PMN to migrate (Figure 7, insert). It is also important to note that none of the concentrations used from EE or fEA affected cell viability (data not shown).

### 2.7. T. granulosa EE and fEA Inhibited TNF-α and IL-1β Gene Expression

**Figure 8:**
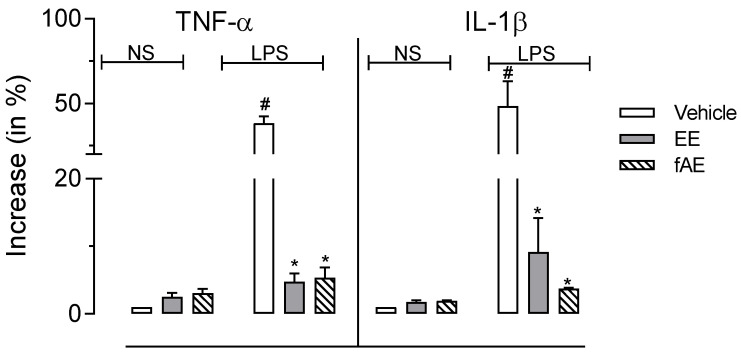
Effects of EE and fEA from *T. granulosa* in TNF-α, IL-1β gene expression. Freshly isolated human polymorphonuclear cells (PMNs) were preincubated with EE or fEA (30 µg/mL) in non-activated (NS) or activated with lipopolysaccharide (LPS, 1 µg/mL). Gene expression for each cytokine was evaluated through RT-PCR. Results show fold increases in gene expression. The statistical significance was calculated using ANOVA followed by the post-test of Newman–Keuls. * *p* < 0.05 when comparing cells that were pre-treated with EE or fEA (30 µg/mL) to cells that received only LPS. # *p* < 0.05 when comparing LPS-activated cells with non-activated cells (NS) receiving vehicle.

Analyses of gene expression were performed through RT-PCR in isolated PMNs previously incubated with EE or fEA activated with LPS. Activation of PMNs with LPS leads to a drastic increase in gene levels of both cytokines, a 38-fold and a 48-fold increase for TNF-α and IL-1β, respectively. The preincubation of PMNs with EE or fEA (30 µg/mL) did not affect the gene levels of both cytokines. On the other hand, both EE and fEA significantly reduced the levels of gene expression (Figure 8).

## 3. Discussion

In the present study, we investigated the possible anti-inflammatory effects of the ethanol extract from the leaves of *T. granulosa*, and its fraction in ethyl acetate.

It has been shown that formalin injection can directly activate nociceptors present in non-myelinated axons, such as C fibers, and poorly myelinated fibers, such as Aδ fibers. It is also able to degranulate mast cells, causing the release of histamine and heparin [22]. These mediators, in turn, activate the nociceptors, also promoting the release of neuropeptides such as substance P and neurokinins, in the peripheral terminals of the primary afferents [23], as well as the excitatory amino acids glutamate and aspartate [24]. Thus, it is believed that this group of chemical mediators seems to be responsible for the transient nociception observed in the first five minutes after formalin injection [25]. Malmberg and Yaksh [26] reported in 1995 that the intraplantar injection of formalin causes a significant increase in glutamate and PGE2 during the second phase of the painful process, whereas the second phase is due to the release of inflammatory mediators acting together in nociceptors and their own local receptors. The involvement of serotonin and bradykinin in both phases was also described [25]. Therefore, the formalin-induced licking model is useful for detecting pain of the non-inflammatory and inflammatory type, corresponding to the first and second phases, respectively. Our data demonstrated that *T. granulosa* EE and fEA did not decrease the first phase of the model, suggesting that they do not have peripheral antinociceptive activity. It is possible that there is a direct effect from substances contained in EE or fEA in nociceptors and/or C fibers, and thus they do not increase the baseline of nociceptors and do not promote antinociceptive activity. Another possibility for the absence of an effect in the 1st phase could be that none of substances present in EE of fEA could affect and/or inhibit some of the algesic mediators produced after formalin injection. However, EE reduced the second phase response, which may indicate an inhibition in the formation and/or release of pro-inflammatory mediators, such as cytokines, eicosanoids, kinins, glutamate, and nitric oxide.

Carrageenan administration in the subcutaneous air pouch induces a multicomplex inflammatory process with characteristics and a course of inflammation similar to that observed in rheumatoid arthritis, with the infiltration of polymorphonuclear leukocytes and release of pro-inflammatory mediators [27]. There is an intense leukocyte migration with a large neutrophil influx, release of chemotactic agents, increase in myeloperoxidase enzyme activity, and the production and liberation of nitric oxide (NO), prostaglandins, histamine, bradykinin, serotonin, and cytokines [28,29]. Our results show a reduction in leukocyte migration. Results obtained in the SAP model complement those obtained in the second phase of formalin-induced licking since both models present an inflammatory profile with involvement in a diversity of inflammatory mediators. Among the hypotheses that may be related to the inhibition of leukocyte migration, we can highlight the inhibition of the production and/or release of pro-inflammatory substances related to leukocyte chemotaxis. This explanation is based on the observation that EE and fEA drastically reduced cytokines (TNF-α, IL-1β, and IFN-γ) production. Some studies have demonstrated that TNF-α can induce the expression of adhesion molecules, such as E-selectin, ICAM-1, and VCAM-1 [30]. IFN-γ has several immunoregulatory effects, including the activation and stimulation of macrophages to release reactive oxygen species. This cytokine increases vascular permeability, thus playing a crucial role in the inflammatory process. Other effects of IFN-γ include the induction of TNF-α and IL-1β production and synergistic effects with TNF-α [31]. Hereby, it could be postulated that the anti-inflammatory activity of the fEA may be associated with the inhibition of the production of cytokines in addition to inhibiting the production of IFN-γ, suggesting a synergistic anti-inflammatory effect.

The presence of hispiduloside in fEA and consequently in EE can explain in part their higher activity in reducing cytokine production and release. Flavonoids are well-known cytokine modulators [32]. It is also very important to note that glycosylated flavonoids are cleaved by intestinal enzymes prior to their absorption, making the aglycone absorbed instead, when given orally. Hispidulin, the aglycone of the flavonoid isolated here has previously shown the downregulation of TNF-a and IL-4 [33]. Taken together, these data complement previous data from other assays suggesting fEA to inhibit cytokines production in exudate, thus reducing leukocyte migration.

The effects of NO on the inflammatory response are complex and involve leukocyte chemotaxis, vasodilation, and increased vascular permeability. The reduction in NO production in the SAP may be a consequence of fewer cells that transmigrated to SAP when the fractions were administered, a decrease in the production of cytokines (i.e., TNF-α, IL-1β, and IFN-γ) that can activate migrating cells, or maybe a reduction in the enzyme iNOS expression or activity. Since our data show that EE and fEA reduced NO production, it can be inferred that this reduction may be caused due to some of these mechanisms.

Experiments with human neutrophils focusing on cell viability, migration, and gene expression, clearly showed that the *T. granulosa* EE and fEA significantly decreased their PMNs migration toward C5a or IL-8, and reduced TNF-α and IL-1β gene expression, all consistent with the results obtained in the SAP model. The exact mechanisms by which these anti-inflammatory phenotypes are brought about, the acceleration of neutrophil apoptosis, cannot be ruled out at this point.

To the best of our knowledge, this work is the first to describe and identify the effects of *T. granulosa* on acute inflammatory models.

## 4. Materials and Methods

### 4.1. Plant Material and Extraction

*Tibouchina granulosa* leaves were collected in Vital Brazil Institute’s farm in Cachoeiras de Macacu, Niteroi, Brazil in October 2017. The material was identified by Dr. Rosana C. Lopes, and a voucher specimen was deposited at the Herbarium of the Biology Institute/UFRJ (number 37,904). Leaves (1.5 kg) were dried at 50 °C, and ground (IKA, model A11BS1, Staufen, Germany). Leaves were extracted for 72 h in ethanol (10 L) using a Soxhlet apparatus. Ethanol extract (EE) was evaporated using a rotary evaporator (IKA, model RV-8) for 40 h (at 40 °C), resulting in 150 g (10% yield). EE (100 g) was submitted to liquid–liquid extraction with 3 L of ethyl acetate, resulting in the fraction in ethyl acetate (fEA, 25.99 g).

### 4.2. Reagents

Formalin was obtained from Merck Inc. (Rio de Janeiro, Brazil). Carrageenan, acetylsalicylic acid (ASA), and dexamethasone were purchased from Sigma Aldrich (St. Louis, MO, USA). All drugs were prepared just before their use and dissolved in saline (NaCl 0.9%). The anesthetics used, ketamine and xylazine (112.5 mg/kg and 7.5 mg/kg, respectively), were dissolved in saline and were purchased from Ceva Ltd. (Paulínea, Brazil). Other solvents used in the extraction and fractionation processes were purchased from TEDIA (Fairfield, OH, USA).

### 4.3. Animals

Female Swiss Webster mice (18–25 g) were donated by Vital Brazil Institute (Niteroi, Brazil). Animals were housed in a room (12 h light/dark cycle, 22 ± 2 °C, 60–80% humidity), with food and water ad libitum. The experiments followed the principles and guidelines adopted by the Brazilian College of Animal Experimentation (COBEA), approved by the Biomedical Science Institute/UFRJ, Ethical Committee for Animal Research, and received the number DFBCICB015-04/16.

### 4.4. fEA Chemical Investigation

fEA was submitted to a silica gel column chromatography (26.0 cm × 3.0 cm), using dichloromethane, gradients of dichloromethane: ethyl acetate, ethyl acetate, gradients of ethyl acetate: methanol, and methanol. Fractions (170) of 10 mL were collected. Fractions 114–128 eluted with mixtures of ethyl acetate (methanol 20% and 30%) represented the fraction containing the compound of interest. After its purification through preparative TLC, the compound was identified as hispiduloside (hispidulin 7-O-D-glucoside), employing ^1^H- and ^13^C-NMR.

#### 4.4.1. HPLC-DAD Quantification

The HPLC system (Waters, Milford, MA, USA) was used in conjunction with Waters 1525 binary HPLC pump, Waters 2487 dual λ absorbance detector, Waters 717 plus autosampler, and breeze software program (Camberley, UK). A reverse-phase Thermo C18 column (250 × 4.6 mm, 5 μm) was used.

Samples and standard solutions (10 µL) were injected in quadruplicate and eluted using the following gradient at a flow of 1.0 mL/min with orthophosphoric acid 0.25% in water (A) and methanol (B): 0 min, 40:60 (A: B); 5 min, 30:70; 10 min, 15:85; 15 min, 35:65; 25 min, 50:50; 30 min, 70:30. The machine was set to detect peaks at 355 nm, and the pressure was at a maximum of 5000 atm. In order to quantify the flavonoid, we used an external standardization with a calibration curve using hispiduloside; the concentration varied between 25 and 300 µg/mL.

#### 4.4.2. Structural Elucidation

Hispiduloside structural elucidation was performed using an Agilent Technology (Santa Clara, CA, USA) 400 NMR apparatus. Hydrogen and carbon 13 spectra were recorded on a BRUKER TOPSPIN 2.1 (^1^H-NMR: 400 and 600 MHz and ^13^C-NMR: 125 MHz) NMR spectrometry system using the Bruker pulse sequence standard (Bruker, Billerica, MA, USA).

### 4.5. Formalin-Induced Licking Behavior

The methodology used for this test was performed in a similar way to the one described by Hunskaar and Hole [34] and adapted by Gomes et al. [35]. Briefly, formalin (20 μL, 2.5% *v*/*v*) was injected into the dorsal surface of the right hind paw, and the time that the animals spent licking the injected paw was recorded in two phases, 0–5 min and 15–30 min after the formalin injection. Mice were orally pre-treated with extract or fEA (10, 30, and 100 mg/kg), ASA (200 mg/kg), or vehicle 60 min before the administration of formalin.

### 4.6. Carrageenan-Induced Inflammation into the Subcutaneous Air Pouch (SAP)

The SAP protocol used was based on that described by Raymundo et al. [36]. Briefly, the mice’s backs were injected subcutaneously with 10 mL of sterile air. Three days later, a new injection with 8 mL of sterile air was made to guarantee the maintenance of the cavity in the animal’s back. On the sixth day, the animals were treated orally with the vehicle, EE or fEA or intraperitoneally with dexamethasone (2.5 mg/kg) 60 min before receiving 1 mL of a sterile carrageenan solution (1%). After 24 h of carrageenan injection, the animals were euthanized with chloral hydrate (1%, i.p.), and the cavity was flushed out with 1 mL of saline. Exudates were collected and centrifuged at 1200 rpm for 10 min at 4 °C, and samples were stored at −20 °C until the quantifications. The total leucocytes cells counts were determined in the exudates using a pocH-100iV Diff (Sysmex, Kobe, Japan) hematology analyzer.

### 4.7. Neutrophils’ Isolation and Stimulation

Polymorphonuclear neutrophils (PMNs) were isolated as described [37]. Briefly, venous blood collected on ethylenediaminetetraacetic acid (EDTA) anticoagulant from healthy volunteers was centrifuged (900 rpm, 15 min), and the resulting platelet-rich plasma was discarded. Leucocytes were obtained following the sedimentation of erythrocytes in 3% dextran. Polymorphonuclear neutrophils were then separated from other leucocytes by centrifugation on a 10 mL lymphocyte separation medium. Contaminating erythrocytes were removed using 20 s of hypotonic lysis. Purified granulocytes (>95% neutrophils, <5% eosinophils) contained less than 0.2% monocytes, were resuspended in HBSS containing 10 mM HEPES (pH 7.4), 1.6 mM Ca^+2^, 10 µg/mL leupeptin, 10 µg/mL aprotinin (antiproteases), and no Mg^+2^. Viability was greater than 98%, as determined by trypan blue dye exclusion. All cells isolation procedures were carried out under sterile conditions at room temperature.

Freshly isolated human neutrophils were resuspended at a density of 10 × 10^6^ (or as indicated) in lysine- and arginine-free RPMI 1640 medium, supplemented with 1% of fetal calf serum (FCS). Adenosine deaminase (0.1 U/mL) was added to cell suspensions, 20 min before stimulation to prevent adenosine accumulation in the extracellular milieu. EE and fEA were dissolved in dimethylsulfoxide and added to cell suspensions 15 min before stimulation. The conditions were non-stimulated (NS) or stimulated with LPS (100 ng/mL), alone or in combination with concentrations of the anti-inflammatory compounds ranging from 0.3 to 30 µg/mL. Cells were incubated for 1 h, 2 h or overnight, as indicated. Incubations were carried out at 37 °C. Cell-free supernatants of stimulated PMNs were stored at −80 °C for later cytokine quantification.

#### Cell Viability—Annexin V/Propidium Iodide Staining

Neutrophils (10 × 10^6^/mL) were placed in a 24-well culture cell plate for analysis of cell viability for 2 h and overnight incubations at 37 °C. After each time point, cells were harvested by transferring them with the complete medium into 1.0 mL reaction tubes. Cells were centrifuged at 12,000 rpm for 3 s and the supernatant was aspirated. Cells were resuspended in 100 μL of staining buffer (Annexin V-FITC staining Kit, BD), and the antibodies were then added, 5 μL of Annexin-V-FITC and 5 μL propidium iodide (PI). The cells were incubated for 15 min at room temperature and protected from light. Finally, 400 µL of staining buffer was added, and the solutions were transferred to appropriate tubes to proceed to sample analysis. Samples were analyzed immediately [38].

### 4.8. Cytokines (IL-1β, TNF-α, IFN-γ), Nitric Oxide (NO), and Proteins Quantifications

Supernatants from the exudates collected from the SAP were used to measure the levels of the cytokines by enzyme-linked immunosorbent assay (ELISA) using the protocol supplied by the manufacturer (B&D, Franklin Lakes, NJ, USA).

To evaluate the nitrate accumulated in SAP, exudates were analyzed according to the method described by Bartholomew [39] and adapted by Raymundo et al. [36], which was followed by the measurement of nitrite according to the Griess reaction [40]. Protein quantification was performed by the BCA method using the protein dosage kit BCA^TM^ (Thermo Fisher Scientific Inc., Whaltman, MA, USA). At the time of the dosage, reagents A (sodium carbonate, sodium bicarbonate, bicinchoninic acid, and sodium tartrate in 0.1 M of sodium hydroxide and B (4% copper sulfate) were mixed in a ratio of 5:1. A volume of 5 µL of the sample was incubated with 195 µL of the BCA reagent for 30 min at 37 °C, and the absorbance was measured in a microplate reader at 562 nm.

### 4.9. RNA Isolation

Cells isolated as described previously were incubated in the concentration of 15 × 10^6^ cells/mL for 1 h, at 37 °C. Total RNA isolated using Trizol (Gibco, Burlington, VT, USA) according to the manufacturer’s protocol. Briefly, cells were mixed into 1 mL Trizol and 200 µL of chloroform were added. After mixing, samples were centrifuged at 12,000 rpm for 15 min, at 4 °C. The aqueous phase was mixed with 250 µL of isopropanol, held at room temperature for 10 min and then centrifuged at 12,000 rpm for 10 min at 4 °C. Supernatants were discarded, and the precipitated RNA pellets were washed twice using 1 mL of ethanol 75% followed by centrifugation in 9000 rpm for 5 min at 4 °C. Pellets were allowed to air-dry for 5 min and resuspended in RNAse-free water. Total mRNA was quantitated using the TECAN reader (Infinite M200, Männedorf, Switzerland).

#### cDNA Reverse Transcription and Real-Time PCR

First-strand cDNA synthesis was performed with 1 µg of total RNA with the Transcriptor First Strand cDNA Synthesis Kit (Roche, Basel, Switzerland) under manufacturer-recommended conditions, using 500 ng of random hexamer primers. Amplification of cDNA was performed in a real-time PCR rotor-Gene Q (Qiagen, Hilden, Germany) operated with Rotor-Gene software version 2.3.1. Each sample consisted of 1 µL of cDNA, 0.2 mM dNTP, 500 nM of primers, 0.1 U of TaqDNA polymerase (Roche Diagnostics, Indianapolis, IN, USA) and SYBR Green I dye (Invitrogen; 1:30,000 dilution) in a reaction volume of 20 µL. Amplification was achieved using 30 cycles of 94 °C (17 s), 58 °C (25 s) and 72 °C steps (25 s) each. Reaction specificity was ascertained after each amplification using the Melt^®^ procedure (60–99 °C, 1 °C/4 s), according to the manufacturer’s protocol. The difference in expression level was determined by normalization to the expression of ACTB as previously described [41]. The human primers used are listed in Table 1.

### 4.10. Neutrophil Chemotaxis Evaluation

PMNs (1.5 × 10^7^ cells/mL) chemotaxis was evaluated as described by Mills et al. [42]. Cell migration was measured with a microplate fluorescence reader (FL600; Bio-Tek Instruments, Winooski, VT, USA) with bottom-read configuration (excitation 485 nm; emission 530 nm).

### 4.11. Statistical Analysis

Results are expressed as the mean ± standard deviation (S.D.). Statistical significance was calculated by analysis of variance (ANOVA) followed by Newman’s post-test. *p* values less than 0.05 (* *p* < 0.05) were considered significant. In vivo, experimental groups were composed of 6–8 animals. For in vitro assays, each protocol was repeated at least for three different days (*n* = 3), and each group test was performed in triplicate.

## 5. Conclusions

Our results evidence the presence of hispiduloside as the main constituent in the ethyl acetate fraction (fEA) of *T. granulosa*. The ethanol extract and fEA from leaves of *T. granulosa* presented a significant effect, reducing the inflammatory parameters. Taken together, our data suggest *T. granulosa* as a plant that presents anti-inflammatory potential.

## Figures and Tables

**Table 1:** Human primers sequences.

Target		Sequence
ACTB	F	5′-CAAATGCTTCTAGGTGGACT-3′
	R	5′-GCTGTCACCTTCACCGTTC-3′
TNF	F	5′-AGCCATGTTGTAGCAAACC-3′
	R	5′-TGAGGTACAGGCCCTCTGAT-3′
IL-1β	F	5′-GGACAAGCTGAGGAAGATGC-3′
	R	5′-TCGTTATCCCATGTGTCGAA-3′

## Data Availability

Data is contained within the article.
